# DNA quality evaluation of formalin-fixed paraffin-embedded heart tissue for DNA methylation array analysis

**DOI:** 10.1038/s41598-023-29120-y

**Published:** 2023-02-03

**Authors:** Mikkel E. Dupont, Steffan N. Christiansen, Stine B. Jacobsen, Marie-Louise Kampmann, Kristine B. Olsen, Jacob Tfelt-Hansen, Jytte Banner, Niels Morling, Jeppe D. Andersen

**Affiliations:** 1 Section of Forensic Genetics, Department of Forensic Medicine, Faculty of Health and Medical Sciences, University of Copenhagen, Copenhagen, Denmark; 2 Section of Forensic Pathology, Department of Forensic Medicine, Faculty of Health and Medical Sciences, University of Copenhagen, Copenhagen, Denmark; 3 Department of Cardiology, Rigshospitalet, Copenhagen University Hospital, Copenhagen, Denmark; 4 Department of Mathematical Sciences, Aalborg University, Aalborg, Denmark

**Keywords:** Epigenetics analysis, Genetic techniques, Epigenetics, Cardiovascular genetics

## Abstract

Archived formalin-fixed and paraffin-embedded (FFPE) heart tissue from autopsied individuals represents an important resource for investigating the DNA methylation of heart tissue of deceased individuals. The DNA quality of FFPE tissue from autopsies may be decreased, affecting the DNA methylation measurements. Therefore, inexpensive screening methods for estimating DNA quality are valuable. We investigated the correlation between the DNA quality of archived FFPE heart tissue examined with the Illumina Infinium HD FFPE QC assay (Infinium QC) and Thermo Fisher’s Quantifiler Trio DNA Quantification kit (QuantifilerTrio), respectively, and the amount of usable DNA methylation data as measured by the probe detection rate (probe DR) obtained with the Illumina Infinium MethylationEPIC array. We observed a high correlation (r^2^ = 0.75; *p* < 10^−11^) between the QuantifilerTrio degradation index, DI, and the amount of usable DNA methylation data analysed with *SeSAMe*, whereas a much weaker correlation was observed between the Infinium QC and *SeSAMe* probe DR (r^2^ = 0.17; *p* < 0.05). Based on the results, QuantifilerTrio DI seems to predict the proportion of usable DNA methylation data analysed with the Illumina Infinium MethylationEPIC array and *SeSAMe* by a linear model: *SeSAMe* probe DR = 0.80–log_10_(DI) × 0.25.

## Introduction

In recent years, the focus on molecular research has improved the diagnosis, prognosis, and treatment of a broad spectrum of diseases.s Large-scale genetic studies have been of great importance for the identification of risk genes. However, DNA sequencing studies do not investigate tissue-specific or environmentally caused gene regulation in diseases^1^.

Environmental factors can lead to somatic mutations of the DNA sequence, as seen in cancer^2^. However, environmental factors may also change the gene expression levels by affecting epigenetic mechanisms such as non-coding RNA, chromatin remodelling, histone modifications, and DNA methylation^1^. The most widely studied epigenetic phenomenon is DNA methylation, the covalent addition of a methyl group to primarily a cytosine nucleotide followed by a guanine nucleotide (CpGs).

Biopsies from surgeries and autopsies have routinely been stored for decades as formalin-fixed and paraffin-embedded (FFPE) tissue^3^. Most pathology and forensic pathology departments have extensive archives of FFPE tissue samples. The archived FFPE samples and clinical data represent enormous resources for tissue-specific disease research. Unfortunately, formalin-fixation of the tissue causes cross-linking between DNA and proteins and damages the DNA leading to short DNA fragments with nicks and other damages^4,5^. Breaking the cross-links requires harsh treatments resulting in further degraded and low-quality DNA for the subsequent analysis^6^.

The Infinium MethylationEPIC assay (EPIC array) (Illumina, Inc., CA, USA) is an array-based hybridization method for interrogating the methylation level at more than 850,000 sites in the genome. The method is widely used for DNA methylation analysis because of the high number of examined methylation sites for a reasonable price^7^. To assess the DNA quality of the samples before the analysis with the EPIC array, Illumina developed a quantitative PCR (qPCR) based DNA quality assessment kit, the Infinium FFPE QC assay (Infinium QC) (Illumina, Inc., CA, USA). EPIC array analyses are relatively costly experiments in person-hours and reagents compared to simple qPCR assays. Therefore, evaluating the DNA quality of tissue samples before EPIC array analysis is highly recommended. The QuantifilerTrio DNA Quantification Kit (QuantifilerTrio) (Thermo Fisher Scientific, Waltham, MA, USA) is another simple and inexpensive qPCR-based assay commonly used in forensic genetic laboratories to determine the quantity and quality of human DNA in biological trace samples.

We evaluated the correlation between the DNA quality measures of two qPCR assays, the Infinium QC and the QuantifilerTrio, and the amount of usable EPIC array probe data generated from FFPE heart tissue samples archived for 10–18 years. The DNA methylation data were analysed with the in silico analysis tools *minfi*^8^ and *SeSAMe*^9^. Based on the data obtained with the EPIC array, Infinium QC, and QuantifilerTrio, analysed with *minfi* and *SeSAMe,* we developed prediction models for estimating the numbers of usable DNA methylation probes to evaluate the usability of FFPE samples for DNA methylation examination before carrying out more costly and time-consuming EPIC array examinations.

## Results

### Comparison of probe detection rates with *minfi* and *SeSAMe*

**Figure 1:**
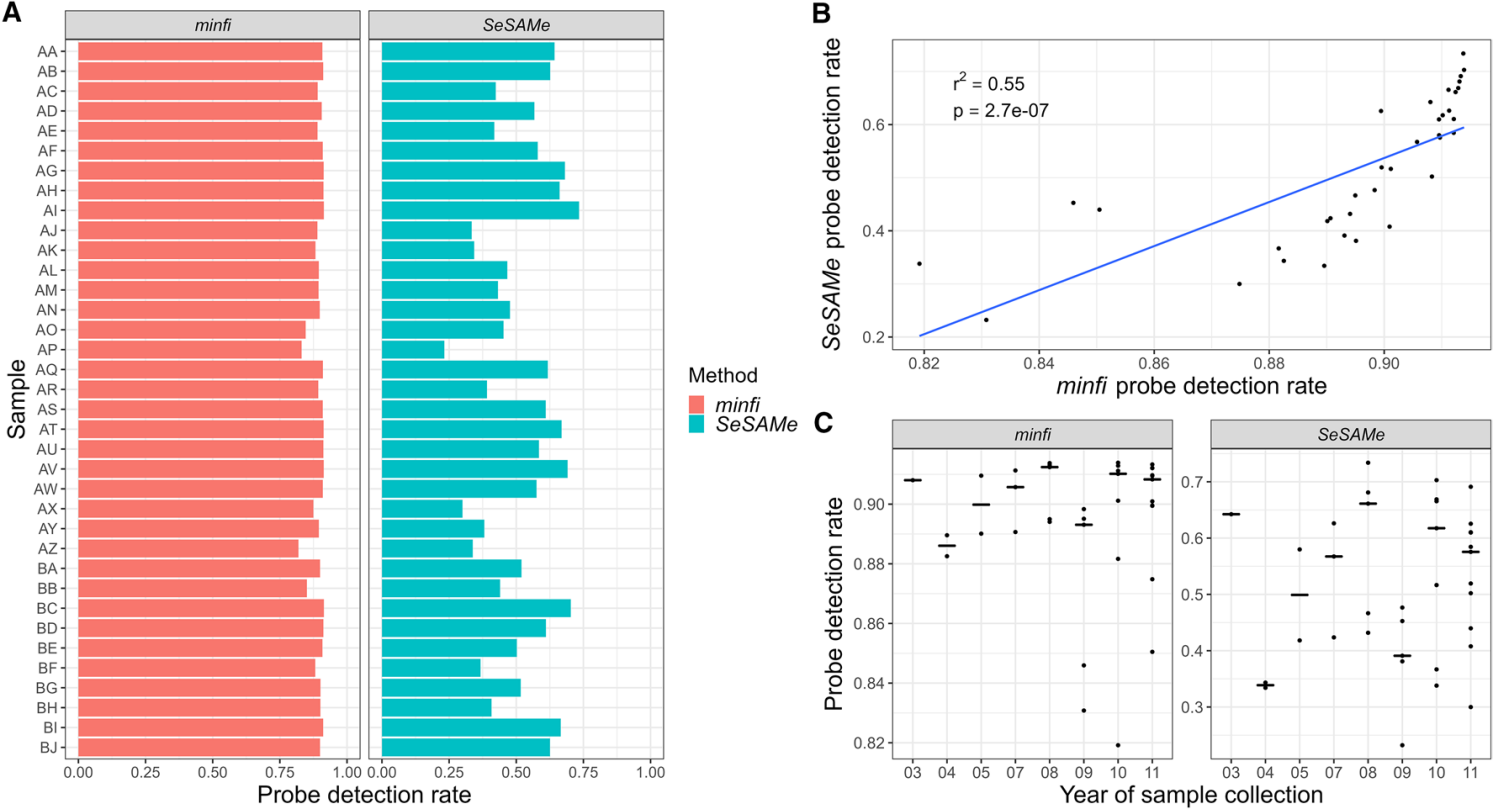
Probe detection rates (probe DR). (**A**) Probe DR of all samples analysed with *minfi* and *SeSAMe*, respectively. (**B**) Correlation between probe DRs analysed with *minfi* and *SeSAMe*. (**C**) Dot plot of probe DRs of samples collected in the years 2003–2011 and analysed with *minfi* (r^2^ < 0.01; *p* > 0.05) and *SeSAMe* (r^2^ = 0.01; *p* > 0.05), respectively, vertical bar indicates median value.

The degrees of DNA methylation of 36 archived FFPE heart tissue samples from the years 2003–2011 were analysed with the EPIC array, and the raw data were analysed with *minfi* and *SeSAMe*. *minfi* discarded 8.6–18.1% of the 862,927 CpG probes, while *SeSAMe* discarded 26.6–76.8% (Fig. 1A). Despite the different probe detection rates (probe DR) of *minfi* and *SeSAMe*, the probe DR correlation between *minfi* and *SeSAMe* data was r^2^ = 0.55 (*p* = 2.7 × 10^−7^) (Fig. 1B). No statistically significant correlation between FFPE storage time and probe DR was observed: *minfi*: r^2^ < 0.01 (*p* > 0.05), and *SeSAMe*: r^2^ = 0.01 (*p* > 0.05) (Fig. 1C).

### Characterization of the probes failing QC with *minfi* and *SeSAMe*

The *minfi* pipeline removed 148,514 probes, while the *SeSAMe* pipeline removed 619,992 probes. With *minfi*, 58,251 (39%) probes failed uniquely in one of the 36 samples, while 20 (0.013%) failed in all 36 samples. With *SeSAMe*, 58.329 (9,4%) probes failed in one of the 36 samples, while 68,393 (11.0%) probes failed in all 36 samples (Supplementary Table S2). Of the probes failing in at least one sample with *minfi*, 133,248 (89.7%) also failed in at least one sample using the *SeSAMe* pipeline, and 49,806 (33.5%) probes failed in all 36 samples with the *SeSAMe* pipeline. When analyzing the samples with *minfi*, the odds was 3.91 times higher (*p* < 2.2 × 10^−16^) for a Type II probe to fail compared to a Type I probe, and it was 4.71 times the odds (*p* < 2.2 × 10^−16^) for a probe annotated to an Open Sea region to fail compared to a probe annotated to a CpG Island region. When analyzing the samples with *SeSAMe*, it was 1.51 times the odds (*p* < 2.2 × 10^−16^) for a Type I probe to fail compared to a Type II probe, and it was 2.69 times the odds (*p* < 2.2 × 10^−16^) for a probe annotated to an Open Sea region to fail compared to a probe annotated to a CpG island region (Supplementary Tables S3 and S4).

With SeSAMe, we also observed differences in fractions of failing probes between samples of high DNA quality (defined as the lower quantile of samples based on DI) and samples of low DNA quality (defined as samples with DI above the upper quantile). We found a statistically significantly higher fraction of failing Type II probes (*p* = 8.2 × 10^−5^) in samples of high quality compared to that of samples with low quality. Furthermore, we observed that samples of high DNA quality had a lower fraction of failing probes annotated to CpG Island region (*p* = 4.0 × 10^−3^) than the low DNA quality samples. For the probes failing with minfi, no statistically significant difference was found between the samples of high and low quality (Supplementary Table S5).

### Probe detection rates with Infinium QC

**Figure 2:**
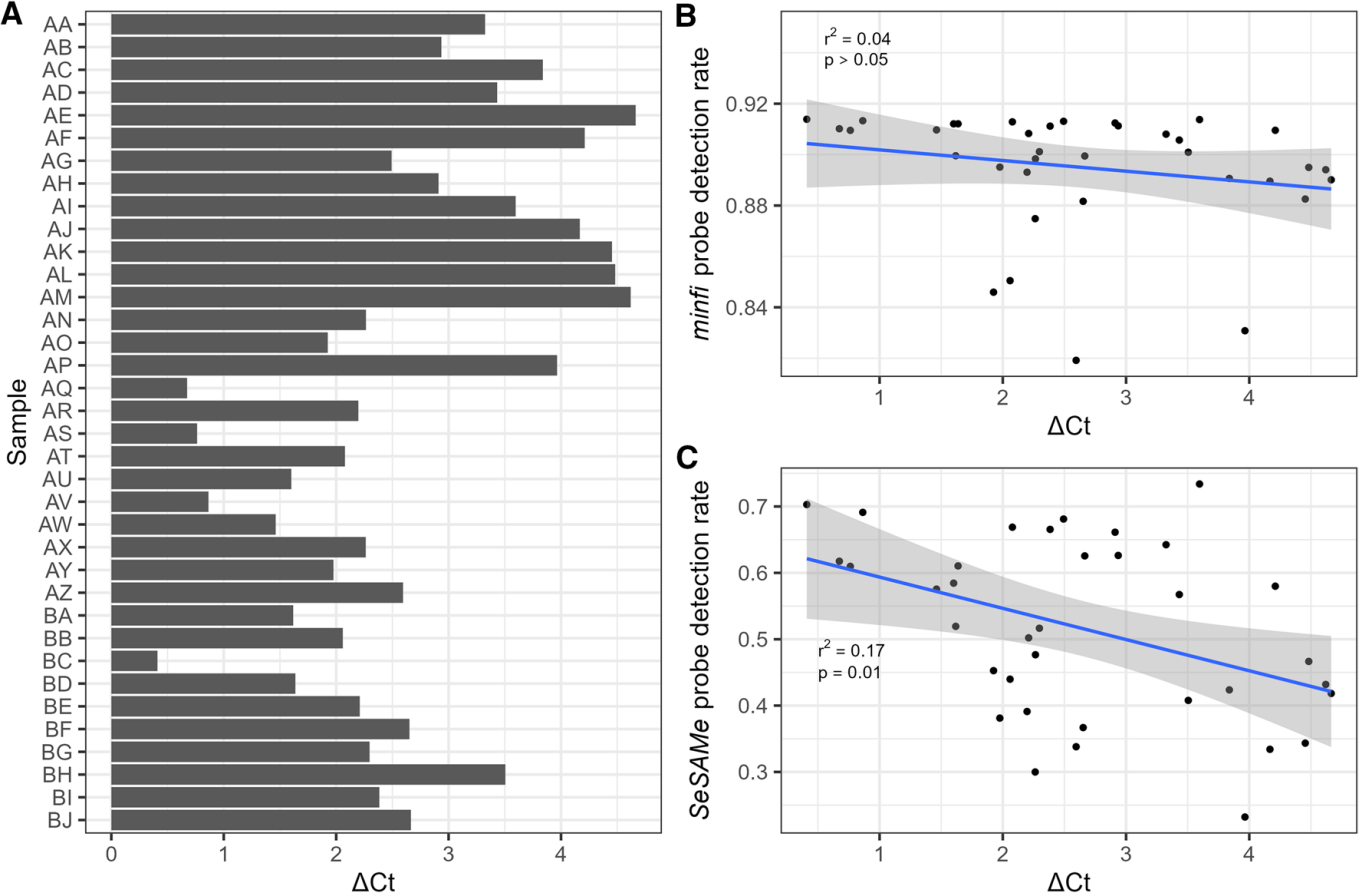
Infinium QC measurements of the DNA quality (ΔCt). (**A**) ΔCt values of each sample. (**B**) Correlation between ΔCt and *minfi* probe DR. The equation of the best fitting linear model was: $minfi \;{\text{probe DR }} = { }0.91{ }{-}{ }\Delta {\text{Ct*}}0.004$. (**C**) Correlation between ΔCt and *SeSAMe* probe DR. The equation of the best fitting linear model was: $SeSAMe\; {\text{probe DR}} = { }0.64{ }{-}{ }\Delta {\text{Ct*}}0.05$.The grey areas in (**B**) and (**C**) indicate the 95% confidence intervals.

The DNA quality measures with Infinium QC, ΔCt, ranged from 0.41 to 4.66 (Fig. 2A and Supplementary Table S1). The larger the ΔCt, the poorer the quality of the DNA. The correlation between ΔCt and probe DR analysed with *minfi* was r^2^ = 0.04 (*p* > 0.05), and with *SeSAMe* r^2^ = 0.17 (*p* = 0.01) (Table 1, Fig. 2B and C). The linear models for prediction of the probe DR were: $minfi \;{\text{probe DR }} = { }0.91{ }{-}\Delta {\text{Ct*}}0.004$ and $SeSAMe \;{\text{probe DR}} = { }0.64{ }{-}\Delta {\text{Ct*}}0.05$.

**Table 1:** Correlation coefficients (r^2^) between probe DR and Infinium QC ΔCt and Quantifiler Trio DI.

	*Minfi*		*SeSAMe*	
	r^2^	*p*	r^2^	*p*
Infinium ΔCt	0.04	> 0.05	0.17	= 0.01
Quantifler Trio DI	0.43	= 1.3 × 10^−4^	0.75	= 8.7 × 10^−12^

**Figure 3:**
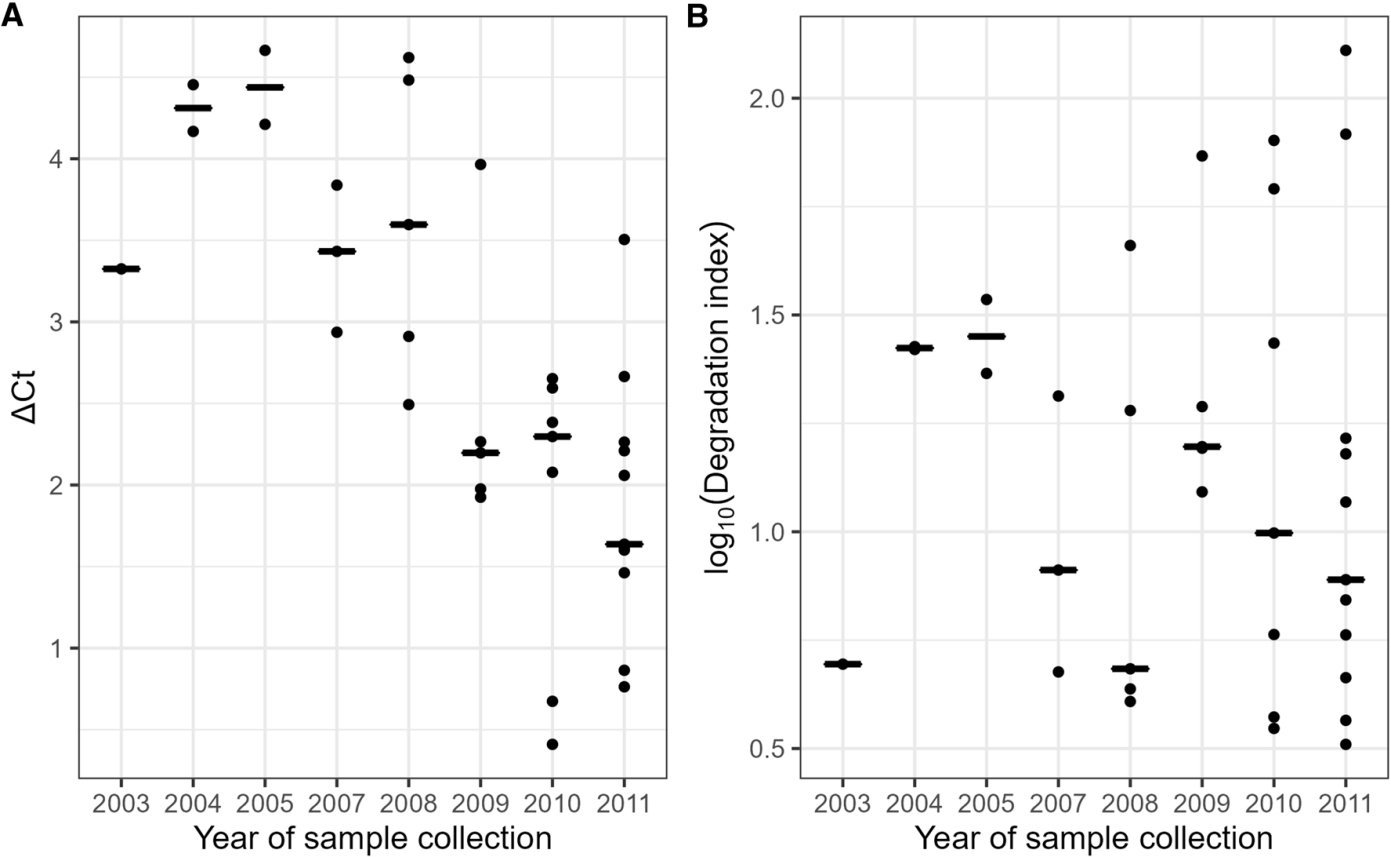
Dot plots of qualitative measurements and storage time, vertical bar indicates median value. (**A**) Infinium QC ΔCt (r^2^ = 0.50; *p* < 10^−5^). (**B**) QuantifilerTrio DI (r^2^ < 0.01; *p* > 0.05).

A statistically significant correlation was found between the storage time and DNA quality measured by ΔCt (r^2^ = 0.50, *p* = 1.4 × 10^−6^) (Fig. 3A).

### Probe detection rates with QuantifilerTrio

**Figure 4:**
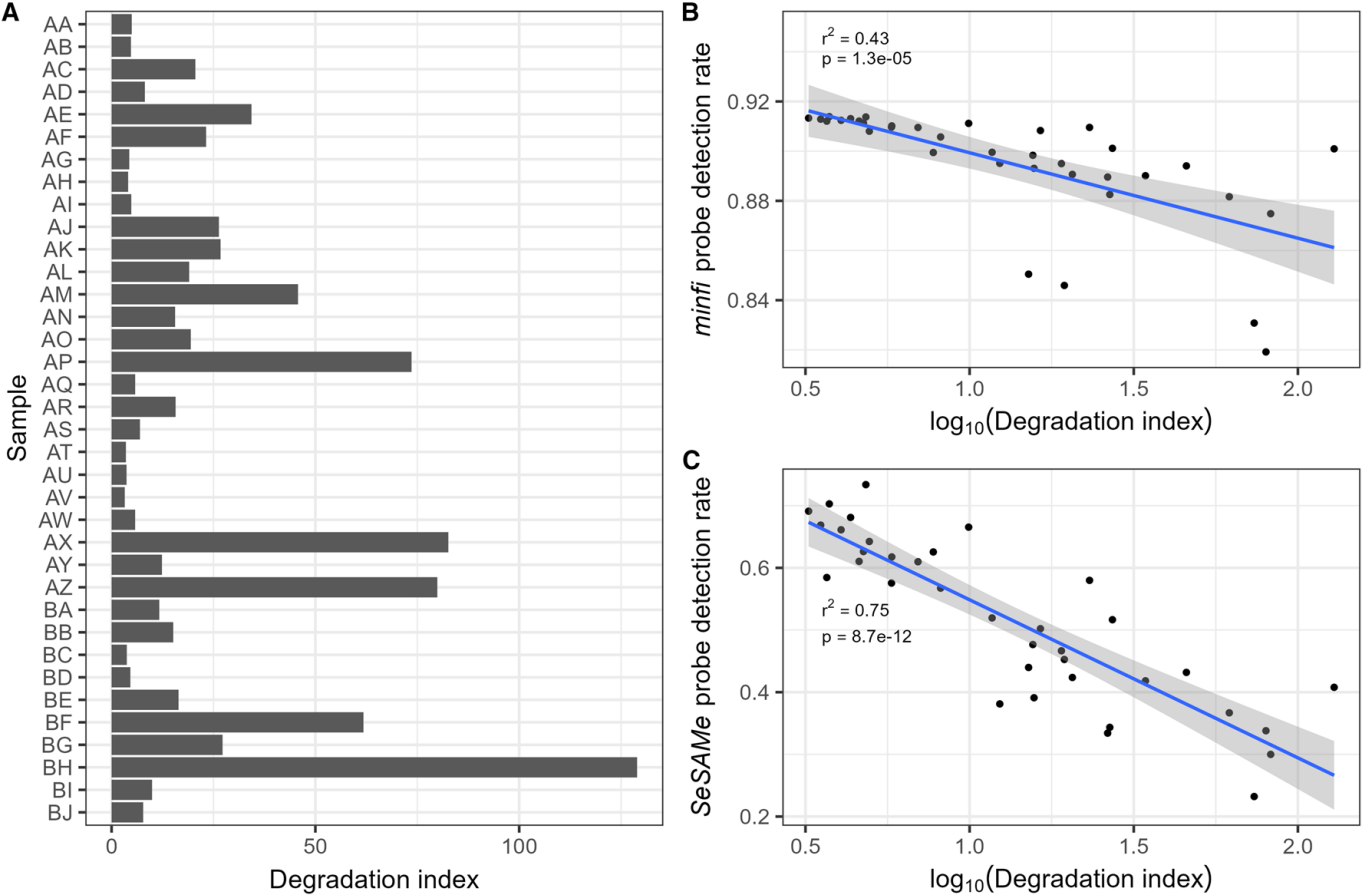
QuantifilerTrio measurements of the DNA quality. (**A**) Degradation index (DI) of each sample. (**B**) Correlation between DI and *minfi* probe DR The equation of the best fitting linear model was: $$minfi\; {\text{probe}} {\text{DR }} = { }0.93{-}{\text{log}}_{10} \left( {{\text{DI}}} \right){*}0.03$$. (**C**) Correlation between DI and *SeSAMe* probe DR. The equation of the best fitting linear model was: $$SeSAMe\; {\text{probe DR }} = { }0.80{-}{\text{log}}_{10} \left( {{\text{DI}}} \right){*}$$$$0.25$$. The grey areas in (**B**) and (**C**) indicate the 95% confidence intervals.

The DNA quality measure of QuantifilerTrio, DI, ranged from 5 to 130 (Fig. 4A and Supplementary Table S1). Larger DNA fragments are more susceptible to degradation than smaller fragments. Thus, the greater the degradation level, the larger the DI. The correlation between log_10_(DI) and probe DR analysed with *minfi* was r^2^ = 0.43 (*p* = 1.3 × 10^−4^), and r^2^ = 0.75 (*p* = 8.7 × 10^−12^) with *SeSAMe* (Table 1, Fig. 4B and C). The linear models for the relationship between probe DRs and DI were: $minfi \;{\text{probe}} {\text{DR }} = { }0.93{ }{-}{\text{ log}}_{10} \left( {{\text{DI}}} \right){*}0.03$, and $SeSAMe \;{\text{probe DR }} = { }0.80{ }{-}{\text{ log}}_{10} \left( {{\text{DI}}} \right){*}0.25$. We did not find any statistically significant correlation between storage time and DNA quality measured as log_10_(DI) (r^2^ < 0.01, *p* > 0.05) (Fig. 3B).

### Prediction of probe detection rates

The linear regression models presented above can be used to predict the probe DR. Heart tissue samples with Infinium QC ΔCt of, e.g., five are predicted to have a mean *minfi* probe DR of 0.89 (0.87–0.90, 95% CI), corresponding to 766,372 usable probes, and a mean *SeSAMe* probe DR of 0.41 (0.31–0.50, 95% CI), corresponding to 351,024 usable probes (Table 2). Heart tissue samples with a ΔCt of, e.g., two are predicted to have a mean *minfi* probe DR of 0.90 (0.89–0.91, 95% CI), corresponding to 777,270 usable probes, and a mean *SeSAMe* probe DR of 0.55 (0.50–0.59, 95% CI), corresponding to 473,265 usable probes.

**Table 2:** Predicted probe detection rates and numbers of detected probes with Infinium QC ΔCt = 5 and QuantifilerTrio DI = 5 (samples of moderate quality).

		Predicted probe detection rate (DR)				Predicted no. of detected probes	
Quality method	Quality value	*minfi*		*SeSAMe*		*minfi*	*SeSAMe*
		DR	95% CI	DR	95% CI	N	N
Infinium ΔCt	ΔCt = 5	0.89	0.87–0.90	0.41	0.31–0.50	766,372	351,024
Quantifler Trio DI	DI = 5	0.91	0.90–0.92	0.63	0.59–0.66	787,686	541,313

Tissue samples of moderate quality with a QuantifilerTrio DI of, e.g., five are predicted to have a mean *minfi* probe DR of 0.91 (0.90–0.92, 95% CI), corresponding to 787,686 usable probes and a mean *SeSAMe* probe DR of 0.63 (0.59–0.66, 95% CI), corresponding to 541,313 usable probes (Table 2). Tissue samples of low quality with a DI of, e.g., 25 are predicted to have a mean *minfi* probe DR of 0.89 (0.88–0.89, 95% CI), corresponding to 766,869 usable probes, and a mean *SeSAMe* probe DR of 0.45 (0.42–0.47, 95% CI), corresponding to 387,419 usable probes.

## Discussion

This study aimed to examine whether the simple, inexpensive DNA quality screening methods Infinium QC and QuantifilerTrio could be used to estimate the quality of the EPIC methylation array data of archived FFPE tissue samples. The EPIC array investigates more than 850,000 CpG sites genome-wide and is rather costly and time-consuming. Thus, it is valuable to assess if the DNA to be examined is of sufficient quality for a successful examination with the expensive EPIC array. DNA quality assessment methods, including the two qPCR-based methods Infinium QC and QuantifilerTrio, are relatively inexpensive compared with the EPIC array. Illumina recommends using the Infinium QC for quality evaluation of FFPE tissue samples before EPIC array experiments. QuantifilerTrio is routinely used in many forensic genetic laboratories to ensure the accuracy and reproducibility of DNA typing. An inverse relationship has been demonstrated between qPCR assayed amplifiable DNA levels, the Ct value, and multiplex PCR SNP mini-sequencing success of FFPE tissue stored for 9–25 years^10^.

We analysed the EPIC methylation array data with the commonly used in silico analysis tools *minfi*^8^ and *SeSAMe*^9^. *SeSAMe* is more restrictive than *minfi,* removing approx. 100,000 more probes than *minfi* using default settings. These probes are removed partly due non-unique mapping as described previously. *SeSAMe* removes particularly data that are technically questionable or flawed. Despite this difference, the probe DRs of *minfi* and *SeSAMe* were correlated with r^2^ = 0.55 (*p* < 10^−6^).

We used the probe DR as the measurement for usable DNA methylation data. The probe DR is strongly influenced by the analysis method and the parameter settings. We used the standard settings of *minfi* and *SeSAMe*. Both with *minfi* and *SeSAMe*, probes annotated to Open Sea regions had statistically significantly (*p* < 2.2 × 10^16^) higher odds of failing than probes annotated to CpG Islands. With *minfi*, Type II probes had higher odds of failing than Type I probes (*p* < 2.2 × 10^−16^), whereas the opposite was observed with *SeSAMe*, where Type I probes had higher odds of failing failed compared to Type II probes (*p* < 2.2 × 10^−16^). With *SeSAMe*, we found that the fraction of failing Type II probes was statistically significantly higher (*p* = 8.2 × 10^−5^) in high DNA quality samples (low DI) compared to that found in the low quality samples (high DI).

We did not find any statistically significant correlation between storage time and the amount of usable DNA methylation data. There was a statistically significant correlation between storage time and Infinium QC ΔCt, but not with QuantifilerTrio DI. The inconsistent results may be due to the relatively short time between sample collection and analysis. It could also be due to the varying post-mortem DNA degradation before the forensic autopsies^11^. The signs of body and tissue decomposition at autopsy before formalin fixation may give a rough estimate of the tissue’s suitability for DNA methylation analysis with the EPIC array. However, the information is not always readily available, particularly not for blocks of old FFPE tissue taken under unknown circumstances. We could not perform this kind of analysis because all heart tissue samples were taken from deceased individuals with no or very little clinical evidence of decomposition.

The probe DR measures the proportion of the investigated probes with sufficient quality. The correlation between the Infinium FFPE QC ΔCt and the probe DR when analysed with *minfi* was r^2^ = 0.04 (*p* > 0.05), and when analysed with *SeSAMe* r^2^ = 0.17 (*p* < 0.05) (Table 1). The correlation between the QuantifilerTrio DI and the probe DR when analysed with *minfi* was r^2^ = 0.43 (*p* < 0.05), and when analysed with *SeSAMe* r^2^ = 0.75 (*p* < 0.05) (Table 1). QuantifilerTrio uses two primer sets, amplifying a short and a long fragment and a dilution series of a DNA standard for DNA quantification. The Infinium QC uses a simple setup comparing the threshold cycle number of a qPCR with a single primer set of the sample to that of a positive control DNA sample. This may be the reason for the difference in efficiency, although other factors, like differences in the chemical formulation of the buffers, may be important.

Based on our data, we established regression models for predicting probe detection rates with Infinium QC ΔCt and QuantifilerTrio DI (Fig. 4). The prediction with QuantifilerTrio of the probe DR analysed with *SeSAMe* performed best (r^2^ = 0.75; *p* = 8.7 × 10^−12^) while the prediction with Infinium QC with *SeSAMe* data was less informative (r^2^ = 0.17; *p* < 0.05). Illumina states that a ΔCt below five is sufficient for successful EPIC array analyses of FFPE tissue^11^. According to the prediction model of Illumina QC, a ΔCt = 5 would lead to a mean probe DR of 0.41 with *SeSAMe* data. When analysing the DNA methylation of fresh auricle tissue from cardiac surgery with the EPIC array using standard settings, we found that the average probe DR with *minfi* data was 0.91, and 0.86 with *SeSAMe* data (publication in preparation). These probe DR values correspond to those obtained with fresh auricular tissue with a Quantifiler DI close to 1. We previously decided to only examine FFPE heart tissue if the Illumina QC ΔCt ≤ 2 (unpublished). The decrease in QuantifilerTrio DI was associated with a larger decrease in probe DR for *SeSAMe* than *minfi* data, possibly reflecting that *SeSAMe’*s data analysis algorithm is more conservative than *minfi’*s. Other tools and pipelines for analysing DNA methylation array data have been published^12–15^. We expect the analyses with these tools will show similar correlations between the probe DR and the quality measures obtained with Infinium QC and QuantifilerTrio.

## Conclusions

The DI of QuantifilerTrio, frequently used in forensic DNA typing of crime scene samples with compromised DNA, correlates better than the Infinium QC ΔCt with the proportion of usable EPIC array methylation probe data when examining FFPE tissue. The relatively cheap QuantifilerTrio performed better for pre-screening of archived FFPE tissue samples before examination with the more expensive EPIC array. A prediction model for the success of DNA methylation examination based on QuantifilerTrio results is presented.

## Materials and methods

### Ethics

The study conformed to the Declaration of Helsinki and was approved by the Committees on Health Research Ethics in the Capital Region of Denmark (H-2-2012-017 and H-190084051). The biobank with the heart tissue samples is registered at the University of Copenhagen’s joint records of processing of personal data in research projects and biobanks (514-0725/22-3000), complying with the General Data Protection Regulation (Regulation (EU) 2016/679).

### FFPE heart tissue, ethics, and DNA extraction

*Heart tissue* was collected in the period 2003–2011 from deceased individuals autopsied at the Section of Forensic Pathology, Department of Forensic Medicine, Faculty of Health and Medical Sciences, University of Copenhagen, Denmark (Supplementary Table S1). The heart tissue was taken from deceased individuals with no or very little clinical evidence of tissue decomposition. After the autopsy, the heart tissue was fixed in 4% buffered formaldehyde (10% buffered formalin) for 48 h and embedded in paraffin using an Excelsior Tissue Processor (Thermo Fisher Scientific, Waltham, MA, USA), and archived at room temperature.

### DNA extraction

DNA was extracted from five 20 µm tissue sections of each sample using the QIAamp DNA FFPE Tissue Kit (Qiagen) according to the manufacturer’s protocol with the modification of increasing the incubation time with proteinase K from 4 h to 16–20 h. The DNA concentration was measured using a Qubit 2.0 Fluorometer (Thermo Fisher Scientific, Waltham, MA, USA).

### DNA methylation analysis

A total of 500 ng DNA was bisulfite-treated using the EZ DNA Methylation kit (Zymo Research Corp) following the manufacturer’s protocol and eluted in 10 µL elution buffer. The eluate was prepared with the Infinium HD FFPE DNA Restore kit (Illumina, Inc., CA, USA) according to the manufacturer’s protocol to repair the bisulfite-treated DNA. The level of DNA methylation was quantified with the Infinium MethylationEPIC Kit (Illumina, Inc., CA, USA) following the manufacturer’s protocol. The prepared slides were scanned using the iScan System (Illumina Inc., CA, USA).

### DNA quality assessment

*Infinium QC*. The quality of DNA from each sample was assessed with the Infinium HD FFPE QC kit (Illumina, Inc., CA, USA) and an ABI 7900 (Thermo Fisher Scientific, Waltham, MA, USA) following the manufacturer’s protocol. The qPCR cycle thresholds (Ct) were used to calculate the ΔCt = Ct(FFPE)–Ct(QCT), where Ct(FFPE) is the Ct of the FFPE heart sample, and Ct(QCT) is the Ct value of the Quality Control Template DNA (QCT) provided by Illumina. The primer set used is expected to generate an amplicon between 175 and 200 bp in length. Illumina considers DNA from FFPE tissue with a ΔCt below five eligible for further analysis with the EPIC array.

### QuantifilerTrio

The quality of DNA from each sample was assessed with the QuantifilerTrio DNA Quantification kit (Thermo Fisher Scientific, Waltham, MA, USA) and an ABI 7900 (Thermo Fisher Scientific, Waltham, MA, USA) following the manufacturer’s protocol. QuantifilerTrio uses three primer sets (a small (80 bp) and a large (214 bp) autosomal amplicon, a Y-chromosomal amplicon (74 bp), and an internal PCR control amplicon (IPC). The ratio between the smaller and larger autosomal amplicons, called the degradation index (DI), was used to measure the DNA quality. A DI ≤ 1 indicates no degradation, a DI from 1 to 10 indicates moderate degradation or inhibition of the PCR, and a DI > 10 indicates severe degradation of the DNA or inhibition of the PCR (Thermo Fisher Scientific, Waltham, MA, USA, 2018).

### Data analysis

The data analysis was conducted in the R statistical environment (R version 4.1.1.) using the *tidyverse* package^16^. The raw iScan data (IDAT files) were imported into R using the Bioconductor^17^ packages *minfi*^8^ and *SeSAMe*^9^.

With *minfi*, the beta values were calculated from the red and green colour intensities (RGChannelSet object) with the *getBeta()* function with no normalization. A detection p-value was calculated to indicate the signal quality at a given site. *minfi* calculated the detection p-value by comparing the total signal (methylated + unmethylated) for each probe with the background signal, estimated from the negative control probes. A small p-value indicates a reliable signal, whereas a high p-value indicates a poor-quality signal. Probes with detection *p* ≥ 0.01 were marked as failed and removed. Probes with common SNPs (SNPs found in dbSNP.137CommonSingle database) that may affect the CpG were removed by the function *dropLociWithSnps()* as well as probes know to cross hybridise to multiple places in the genome^7^.

With *SeSAMe*, the beta values were calculated using the function *getBetas(),* applying its own normalization method (*p*-value with out-of-band array hybridization; pOOBAH)). In addition to discarding probes with detection *p* ≥ 0.01, *SeSAMe* also discards probes that hybridize with DNA fragments from loci giving incorrect DNA methylation levels^9^. Using the *SeSAMeQC()* function, the loaded raw IDAT files were quality checked, and the number of removed probes (number_na_cg) for each samples was determined.

We defined the probe detection rate (DR) as:

$$probe detection rate = \frac{total CG probes - removed CG probes}{{total CG probes}}.$$

The IlluminaHumanMethylationEPICanno.ilm10b4.hg19^18^ package was used to annotate probes as Type I or Type II and annotate the probes to CpG Islands and Open Sea regions.

### Statistics

#### Correlation

Pearson’s correlation coefficient of determination (r^2^) was calculated for the pairwise comparisons of probe DRs with Infinium QC ΔCt and QuantifilerTrio DI quality measurements. The plots were made with *ggplot2* in R.

#### Linear regression

The *lm()* function was used to determine the best fitting linear regression model for predicting the probe DR based on (1) the Infinium QC ΔCt data and (2) the best fitting log-linear regression model based on the QuantifilerTrio DI data obtained with *minfi* and *SeSAMe,* respectively.

#### Fisher's exact test

The *fisher.test()* function was used to identify statistically significant differences between the failing and non-failing probes.

#### Wilcoxon rank sum test

The *wilcox.test()* function was used to identify statistically significant differences between the failing probes of the top and bottom quantiles of samples, based on the QuantifilerTrio DI.

### Informed consent

Due to autopsies being performed prior to 1 January 2012, informed consent was waived in accordance with the Danish Health Act and the Consolidated Act no. 1338 of 1 September 2020 on Research Ethics Review of Health Research Projects and Health Data Research Projects.

## Supplementary Information

Supplementary Information.

## Data Availability

The datasets used and/or analysed during the current study available from the corresponding author on reasonable request.
