# Supplementary material for: DNA quality evaluation of formalin-fixed paraffin-embedded heart tissue for DNA methylation array analysis
Source: Sci Rep. 2023 Feb 3;13:2004. doi: 10.1038/s41598-023-29120-y (PMC9898234; doi:10.1038/s41598-023-29120-y)
Supplement: Supplementary file 1 — Supplementary Information. [file 41598_2023_29120_MOESM1_ESM.docx]

**Supplementary information**

**Supplementary Table *S1*.** Sample collection year, *minfi* probe detection rate (DR), *SeSAMe* probe DR, ΔCt, and degradation index (DI).

| Sample | Sample year | *Minfi* probe DR | *SeSAMe* probe DR | ΔCt | DI |
| --- | --- | --- | --- | --- | --- |
| AA | 2003 | 0.993 | 0.644 | 3.32 | 4.95 |
| AB | 2007 | 0.996 | 0.627 | 2.94 | 4.75 |
| AC | 2007 | 0.974 | 0.426 | 3.84 | 20.56 |
| AD | 2007 | 0.990 | 0.569 | 3.43 | 8.16 |
| AE | 2005 | 0.974 | 0.420 | 4.66 | 34.33 |
| AF | 2005 | 0.994 | 0.581 | 4.21 | 23.19 |
| AG | 2008 | 0.998 | 0.682 | 2.49 | 4.34 |
| AH | 2008 | 0.997 | 0.662 | 2.91 | 4.06 |
| AI | 2008 | 0.999 | 0.735 | 3.60 | 4.83 |
| AJ | 2004 | 0.973 | 0.336 | 4.17 | 26.33 |
| AK | 2004 | 0.966 | 0.346 | 4.45 | 26.73 |
| AL | 2008 | 0.979 | 0.468 | 4.48 | 19.04 |
| AM | 2008 | 0.978 | 0.434 | 4.62 | 45.74 |
| AN | 2009 | 0.982 | 0.478 | 2.27 | 15.59 |
| AO | 2009 | 0.926 | 0.454 | 1.93 | 19.44 |
| AP | 2009 | 0.910 | 0.235 | 3.97 | 73.59 |
| AQ | 2010 | 0.995 | 0.619 | 0.67 | 5.79 |
| AR | 2009 | 0.977 | 0.393 | 2.20 | 15.71 |
| AS | 2011 | 0.994 | 0.611 | 0.76 | 6.96 |
| AT | 2010 | 0.998 | 0.670 | 2.08 | 3.52 |
| AU | 2011 | 0.997 | 0.586 | 1.60 | 3.67 |
| AV | 2011 | 0.998 | 0.692 | 0.86 | 3.23 |
| AW | 2011 | 0.995 | 0.577 | 1.46 | 5.78 |
| AX | 2011 | 0.958 | 0.302 | 2.26 | 82.62 |
| AY | 2009 | 0.979 | 0.383 | 1.98 | 12.36 |
| AZ | 2010 | 0.898 | 0.340 | 2.60 | 79.92 |
| BA | 2011 | 0.984 | 0.521 | 1.62 | 11.71 |
| BB | 2011 | 0.931 | 0.442 | 2.06 | 15.13 |
| BC | 2010 | 0.999 | 0.704 | 0.41 | 3.74 |
| BD | 2011 | 0.997 | 0.612 | 1.64 | 4.61 |
| BE | 2011 | 0.993 | 0.504 | 2.21 | 16.43 |
| BF | 2010 | 0.965 | 0.369 | 2.65 | 61.80 |
| BG | 2010 | 0.986 | 0.518 | 2.30 | 27.23 |
| BH | 2011 | 0.985 | 0.410 | 3.51 | 128.93 |
| BI | 2010 | 0.996 | 0.667 | 2.38 | 9.93 |
| BJ | 2011 | 0.983 | 0.627 | 2.67 | 7.75 |

**Supplementary Table *S2*.** Numbers and fractions of failing probes.

| Number of samples | Number of probes failed with the *minfi* pipeline | Fraction of probes failed with the *minfi* pipeline | Number of probes failed with the *SeSAMe* pipeline | Fraction of probes failed with the *SeSAMe* pipeline |
| --- | --- | --- | --- | --- |
| Any | 148,514 | 1.000 | 619,992 | 1.000 |
| 1 | 58,251 | 0.392 | 58,329 | 0.094 |
| 2 | 25,466 | 0.171 | 33,082 | 0.053 |
| 3 | 15,055 | 0.101 | 24,241 | 0.039 |
| 4 | 9,901 | 0.067 | 20,038 | 0.032 |
| 5 | 7,156 | 0.048 | 17,861 | 0.029 |
| 6 | 5,096 | 0.034 | 16,371 | 0.026 |
| 7 | 4,208 | 0.028 | 15,423 | 0.025 |
| 8 | 3,423 | 0.023 | 15,028 | 0.024 |
| 9 | 2,833 | 0.019 | 14,715 | 0.024 |
| 10 | 2,391 | 0.016 | 14,279 | 0.023 |
| 11 | 2,139 | 0.014 | 14,059 | 0.023 |
| 12 | 1,815 | 0.012 | 13,662 | 0.022 |
| 13 | 1,523 | 0.010 | 13,403 | 0.022 |
| 14 | 1,388 | 0.009 | 13,516 | 0.022 |
| 15 | 1,187 | 0.008 | 13,596 | 0.022 |
| 16 | 1,005 | 0.007 | 13,068 | 0.021 |
| 17 | 850 | 0.006 | 12,996 | 0.021 |
| 18 | 760 | 0.005 | 12,942 | 0.021 |
| 19 | 619 | 0.004 | 12,894 | 0.021 |
| 20 | 523 | 0.004 | 12,681 | 0.021 |
| 21 | 464 | 0.003 | 12,429 | 0.020 |
| 22 | 429 | 0.003 | 11,578 | 0.019 |
| 23 | 345 | 0.002 | 11,474 | 0.019 |
| 24 | 275 | 0.002 | 11,170 | 0.018 |
| 25 | 246 | 0.002 | 10,620 | 0.017 |
| 26 | 229 | 0.002 | 10,544 | 0.017 |
| 27 | 190 | 0.001 | 10,400 | 0.017 |
| 28 | 164 | 0.001 | 10,425 | 0.017 |
| 29 | 131 | 0.001 | 10,632 | 0.017 |
| 30 | 101 | 0.001 | 10,758 | 0.017 |
| 31 | 101 | 0.001 | 11,479 | 0.019 |
| 32 | 74 | 0.0005 | 12,239 | 0.020 |
| 33 | 73 | 0.0005 | 13,860 | 0.022 |
| 34 | 48 | 0.0003 | 16,819 | 0.027 |
| 35 | 35 | 0.0002 | 24,998 | 0.040 |
| 36 | 20 | 0.0001 | 68,393 | 0.110 |

**Supplementary Table *S3*.** Fraction of probes of Type I or Type II.

| Probe Type | Type I | Type II |
| --- | --- | --- |
| Fraction of all analyzed probes* (*minfi*) | 0.160 | 0.840 |
| Fraction of probes failed with the *minfi* pipeline | 0.055 | 0.945 |
| Fraction of all analyzed probes* (*SeSAMe*) | 0.160 | 0.840 |
| Fraction of probes failed with the *SeSAMe* pipeline | 0.133 | 0.866 |

*) : Cross-hybridizing probes and common SNPs (SNPs found in the dbSNP.137CommonSingle database) were removed with the *dropLociWithSnps()* function of *minfi*.

**Supplementary Table *S4*.** Fraction of probes grouped by their locations relative to the CpG Islands.

| Relation to CpG island | North Shelf | North Shore | CpG Island | South Shore | South Shelf | Open sea |
| --- | --- | --- | --- | --- | --- | --- |
| Fraction of all analyzed probes* (*minfi*) | 0.036 | 0.980 | 0.192 | 0.084 | 0.033 | 0.557 |
| Fraction of probes failed with the *minfi* pipeline | 0.037 | 0.089 | 0.059 | 0.076 | 0.035 | 0.704 |
| Fraction of all analyzed probes* (*SeSAMe*) | 0.035 | 0.098 | 0.194 | 0.084 | 0.033 | 0.557 |
| Fraction of probes failed with the *SeSAMe* pipeline | 0.034 | 0.100 | 0.170 | 0.086 | 0.032 | 0.578 |

*): Cross-hybridizing probes and common SNPs (SNPs found in the dbSNP.137CommonSingle database) were removed with the *dropLociWithSnps()* function of *minfi*.

**Supplementary Table *S5*.** Fraction of probes failed by *minfi* or *SeSAMe* grouped by probe type or location.

| Sample | *minfi* fraction Type II* | *minfi* fraction Open Sea | *minfi* fraction CpG Island | *SeSAMe* fraction Type II* | *SeSAMe* fraction Open Sea | *SeSAMe* fraction CpG Island |
| --- | --- | --- | --- | --- | --- | --- |
| AA | 0.9635 | 0.8206 | 0.0212 | 0.9328 | 0.6840 | 0.0549 |
| AB | 0.9468 | 0.7579 | 0.0403 | 0.9077 | 0.6044 | 0.1036 |
| AC | 0.9659 | 0.7848 | 0.0172 | 0.8926 | 0.5940 | 0.1402 |
| AD | 0.9524 | 0.7701 | 0.0261 | 0.9022 | 0.5942 | 0.1213 |
| AE | 0.9546 | 0.7837 | 0.0175 | 0.8903 | 0.6994 | 0.1470 |
| AF | 0.9369 | 0.7299 | 0.0429 | 0.8702 | 0.5218 | 0.1843 |
| AG | 0.9509 | 0.7616 | 0.0451 | 0.9223 | 0.6426 | 0.0747 |
| AH | 0.9518 | 0.7927 | 0.0432 | 0.9150 | 0.6651 | 0.0756 |
| AI | 0.9175 | 0.6537 | 0.1184 | 0.8911 | 0.5789 | 0.1126 |
| AJ | 0.9503 | 0.7280 | 0.0432 | 0.8585 | 0.5487 | 0.1907 |
| AK | 0.9597 | 0.7525 | 0.0318 | 0.8658 | 0.5614 | 0.1799 |
| AL | 0.9738 | 0.8060 | 0.0086 | 0.9089 | 0.6364 | 0.1636 |
| AM | 0.9661 | 0.7495 | 0.0341 | 0.8686 | 0.5507 | 0.1825 |
| AN | 0.9173 | 0.6665 | 0.0581 | 0.9000 | 0.5959 | 0.1249 |
| AO | 0.9300 | 0.7284 | 0.0430 | 0.8697 | 0.5606 | 0.1740 |
| AP | 0.9478 | 0.7309 | 0.0406 | 0.8886 | 0.5988 | 0.1434 |
| AQ | 0.9233 | 0.7554 | 0.0268 | 0.9283 | 0.6565 | 0.0678 |
| AR | 0.9422 | 0.7350 | 0.0276 | 0.9422 | 0.7350 | 0.0276 |
| AS | 0.9182 | 0.7319 | 0.0379 | 0.9232 | 0.6397 | 0.0790 |
| AT | 0.9328 | 0.7817 | 0.0455 | 0.9226 | 0.6418 | 0.0720 |
| AU | 0.9248 | 0.7214 | 0.0516 | 0.8994 | 0.5918 | 0.1236 |
| AV | 0.9343 | 0.6727 | 0.0771 | 0.9205 | 0.6276 | 0.0778 |
| AW | 0.9548 | 0.8035 | 0.0186 | 0.9234 | 0.6316 | 0.0928 |
| AX | 0.9228 | 0.6907 | 0.0838 | 0.8556 | 0.5559 | 0.1905 |
| AY | 0.9374 | 0.7032 | 0.0503 | 0.9070 | 0.6145 | 0.1174 |
| AZ | 0.9735 | 0.7871 | 0.0143 | 0.8913 | 0.5964 | 0.1472 |
| BA | 0.9225 | 0.7185 | 0.0511 | 0.8440 | 0.5007 | 0.2151 |
| BB | 0.9602 | 0.7698 | 0.0231 | 0.9001 | 0.6076 | 0.1294 |
| BC | 0.9061 | 0.7509 | 0.0487 | 0.9346 | 0.6743 | 0.0512 |
| BD | 0.8898 | 0.7105 | 0.0615 | 0.9178 | 0.6390 | 0.0884 |
| BE | 0.9382 | 0.7608 | 0.0404 | 0.8796 | 0.5590 | 0.1013 |
| BF | 0.9723 | 0.7871 | 0.0143 | 0.8849 | 0.5827 | 0.1567 |
| BG | 0.8272 | 0.4952 | 0.2611 | 0.8260 | 0.4903 | 0.2380 |
| BH | 0.9209 | 0.6591 | 0.0819 | 0.8479 | 0.5188 | 0.2095 |
| BI | 0.9077 | 0.6907 | 0.0736 | 0.8867 | 0.5867 | 0.1166 |
| BJ | 0.9110 | 0.6971 | 0.0492 | 0.8919 | 0.5843 | 0.1153 |

*Fractions of Type I probes = 1 - fraction of Type II.
